# Group size and mating system predict sex differences in vocal fundamental frequency in anthropoid primates

**DOI:** 10.1038/s41467-023-39535-w

**Published:** 2023-07-10

**Authors:** Toe Aung, Alexander K. Hill, Dana Pfefferle, Edward McLester, James Fuller, Jenna M. Lawrence, Ivan Garcia-Nisa, Rachel L. Kendal, Megan Petersdorf, James P. Higham, Gérard Galat, Adriano R. Lameira, Coren L. Apicella, Claudia Barelli, Mary E. Glenn, Gabriel Ramos-Fernandez, David A. Puts

**Affiliations:** 1 Department of Anthropology, Pennsylvania State University, University Park, PA USA; 2 Psychology and Counseling Department, Immaculata University, Immaculata, PA USA; 3 Department of Anthropology, University of Washington, Seattle, WA USA; 4 Welfare and Cognition Group, Cognitive Neuroscience Laboratory, German Primate Center – Leibniz Institute for Primate Research, Goettingen, Germany & Leibniz-ScienceCampus Primate Cognition, German Primate Center & University of Goettingen, Goettingen, Germany; 5 Department for the Ecology of Animal Societies, Max Planck Institute of Animal Behavior, Konstanz, Germany; 6 Department of Ecology, Evolution, and Environmental Biology, Columbia University, New York, NY USA; 7 Department of Anthropology, Durham University, Durham, UK; 8 Department of Anthropology, New York University, 25 Waverly Place, New York, NY USA; 9 IRD (French National Research Institute for Sustainable Development), Montpellier, France; 10 Department of Psychology, University of Warwick, Coventry, UK; 11 Department of Psychology, University of Pennsylvania, Philadelphia, PA USA; 12 Department of Biology, University of Florence, Sesto Fiorentino, Florence, Italy; 13 Department of Anthropology, California State Polytechnic University Humboldt, Arcata, CA USA; 14 Institute for Research on Applied Mathematics and Systems and C3-Centro de Ciencias de la Complejidad, Universidad Nacional Autonoma de Mexico, Mexico, Mexico City, Mexico

**Keywords:** Sexual selection, Biological anthropology, Animal behaviour, Social evolution, Animal physiology

## Abstract

Vocalizations differ substantially between the sexes in many primates, and low-frequency male vocalizations may be favored by sexual selection because they intimidate rivals and/or attract mates. Sexual dimorphism in fundamental frequency may be more pronounced in species with more intense male mating competition and in those with large group size, where social knowledge is limited and efficient judgment of potential mates and competitors is crucial. These non-mutually exclusive explanations have not been tested simultaneously across primate species. In a sample of vocalizations (*n* = 1914 recordings) across 37 anthropoid species, we investigated whether fundamental frequency dimorphism evolved in association with increased intensity of mating competition (H1), large group size (H2), multilevel social organization (H3), a trade-off against the intensity of sperm competition (H4), and/or poor acoustic habitats (H5), controlling for phylogeny and body size dimorphism. We show that fundamental frequency dimorphism increased in evolutionary transitions towards larger group size and polygyny. Findings suggest that low-frequency male vocalizations in primates may have been driven by selection to win mating opportunities by avoiding costly fights and may be more important in larger groups, where limited social knowledge affords advantages to rapid assessment of status and threat potential via conspicuous secondary sexual characteristics.

## Abstract (web summary)

Sexual dimorphism in the fundamental frequency of primate vocalizations is variable. Here, the authors examine 1914 vocalizations from 37 anthropoid species to find that fundamental frequency dimorphism increased with larger group size and polygyny, due to sexual selection.

## Introduction

Determining why sex differences evolved and vary among primates is critical to understanding the evolution of mating systems and social organization^1^. Sexual dimorphism in body size, skeletal size and shape, dentition, pelage coloration, and ornamentation have been studied extensively^2–6^, but little is known about the evolution of sex differences in primate vocalizations despite the importance of vocal communication^7–9^. Comparative studies have focused on acoustic allometry, i.e. the link between an animal’s body size and the acoustic properties of its vocalizations^10–13^, as well as adaptive explanations (e.g., acoustic variations in response to the degree of sperm competition^14^ and the strength of social bonding^15^). However, these studies have conducted analyses at the species level without distinguishing sex, or within one sex only. Primate larynges, including extralaryngeal appendices^16,17^, are also substantially larger in proportion to body size, more variable in size, and have evolved faster than carnivore larynges^18^, with male primate vocalizations and vocal anatomy often seeming to exaggerate the appearance of body size to perceivers^19–23^. Among polygynous great apes, flanged orangutan and silverback gorilla males, not only have bigger body sizes but also have larger larynges, which produce lower frequencies than non-flanged and black-backed males^24^.

Because of their potential roles in mediating agonistic same-sex contests and/or attracting mates^1,11,20,25–27^, low-frequency male vocalizations may be under positive sexual selection in various primate species (e.g., howler monkeys^14^, guenons^28^, and humans^29–32^). Some evidence suggests that sexual dimorphism in fundamental frequency (*f*_o_) across anthropoid primates increased during evolutionary transitions towards polygyny and decreased during transitions towards monogamy^1^. If sexual dimorphism in *f*_o_ increases with more intense male mating competition (H1: mating competition intensity hypothesis), then male *f*_o_ may decrease relative to female *f*_o_ in species with a more female-biased adult sex ratio, polygynous mating system, and increased male–male intrasexual competition (Supplementary Table 1).

Furthermore, conspicuous sexually selected traits may be particularly important to fitness in species with larger group sizes and multilevel social organization, where social knowledge is limited and efficient evaluation of potential mates and competitors is exigent. Larger groups result in more social interactions and conflicts but lower reliability of signal recognition. Signals for quick assessments of intrinsic quality are more likely to be important in a large group size due to fewer repeated interactions with a given individual, a higher probability of encountering individuals with similar phenotypes, and a greater advantage of distinctiveness as more phenotypes need to be discriminated^5,33–36^. Larger group size has been associated with more visually conspicuous traits^5^ and larger vocal repertoires^15^ in primates. Sexual dimorphism in *f*_o_ may be greater in species with larger group size (H2: group size hypothesis) and multilevel social organizations (H3: social organization hypothesis), in which individuals frequently interact with conspecifics about whom they have limited social knowledge (e.g., Guinea baboons^37^).

More intense sperm competition may also decrease investment in enlarged vocal anatomical structures underlying low-frequency male vocalizations^11,14^ due to a trade-off between investment in pre-copulatory and post-copulatory competitive traits^38,39^. If so, then increased testes volume should predict decreased *f*_o_ dimorphism (H4: sperm competition hypothesis). Compared to those in open, terrestrial habitats, species occupying arboreal habitats face greater visual obstruction and greater attenuation of acoustic signals. Therefore, sexual dimorphism of *f*_o_ might arise due to selection favoring low-frequency calls^40–43^. While terrestrial species are generally expected to have larger sexual dimorphism in body size, body size dimorphism may be constrained in arboreal species for efficient foraging^3^. Thus, selection pressures on vocalization may be particularly important; arboreal species may display greater *f*_o_ dimorphism than terrestrial species for a given body size and degree of body size dimorphism (H5: habitat hypothesis).

**Fig. 1:**
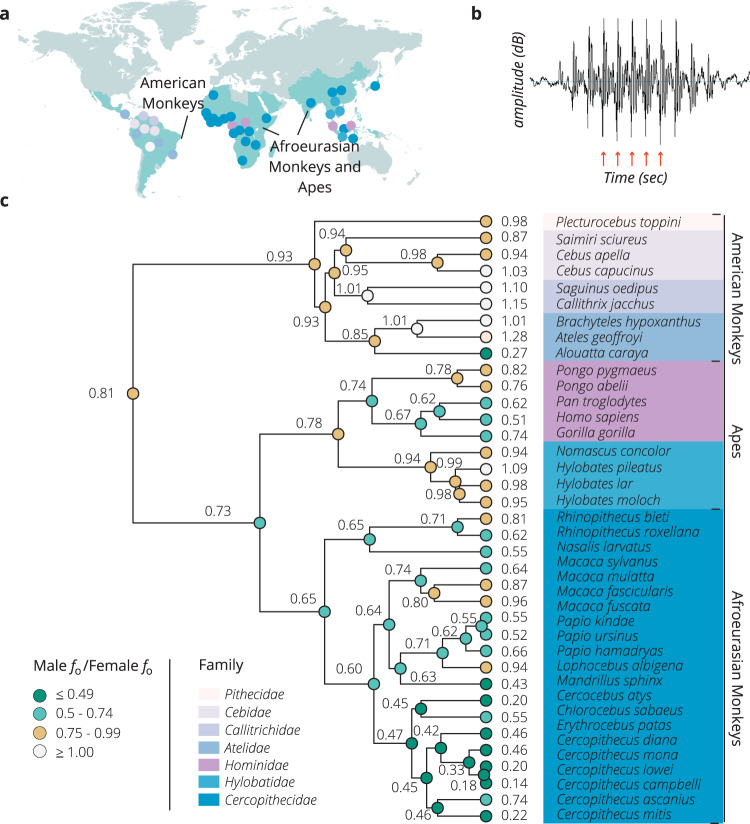
Primate distribution, acoustic measurement, and phylogenetic tree of anthropoid primates for which data were available on at least two recordings of vocalizations from each sex. Panel (**a**) represents species included in our study in relation to the worldwide distribution of nonhuman primate species (green shading). Data were extracted using www.iucnredlist.org and plotted using www.mapchart.net. Panel (**b**) shows a waveform vocalization of a *P. hamadryas* male grunt as an example for calculating fundamental frequency. The amplitude, measured in decibels, represents loudness. Each arrow across time represents a pulse (opening and closing of the vocal folds). The rate of pulses per second equals the fundamental frequency (Hertz). Panel (**c**) shows a phylogenetic tree, constructed using a consensus phylogeny for all species, except three, in our sample from the 10kTrees website (http://10ktrees.fas.harvard.edu/); the phylogenetic estimates of *B. hypoxanthus, P. toppini*, and *P. kindae* were inferred using *B. arachnoides* and *C. moloch* data from the 10kTrees website, and a splitting date of 0.60 million years ago from *P. ursinus*, respectively^74^. Sexual dimorphism (male/female) in fundamental frequency is shown in the column to the left of species names (see Methods for how sexual dimorphism in fundamental frequency is obtained for each species). Inferred ancestral states were reconstructed using the maximum likelihood approach, as implemented in the *fastANC* function in the *phytools* R package, and are shown at nodes on the tree (colored to highlight evolutionary trends). Source data are provided as a Source Data file.

Finally, *f*_o_ dimorphism may be a by-product of selection for greater male size^12,13^, or phylogeny^1,11,20^. We tested these hypotheses in a sample of 1914 vocalizations representing 37 anthropoid species (Fig. 1a). Here, we show that sexual dimorphism in *f*_o_ increases in evolutionary transitions towards a larger group size and polygynous mating system. These findings highlight that selection to win mating opportunities while avoiding costly fights likely contributes to deep male vocalizations in primates, which may be more important in larger groups where social knowledge is limited and rapid assessment of status and threat potential via conspicuous secondary sexual characteristics is crucial.

## Results and discussion

### Predictors of *f*_o_ and *f*_o_ dimorphism

Our analyses confirmed acoustic allometry. Males and females from larger species tend to have relatively lower *f*_o_ values (Supplementary Fig. 1). In a phylogenetic regression model (λ = 0.96) that predicts male and female *f*_o_ values, both larger body mass (*post.mean* = −0.61; *p* < 0.001) and sex predicted differences in *f*_o_, with males having lower *f*_o_ than females (*post.mean* = −0.24; *p* = 0.046). In subsequent phylogenetic analyses that controlled for body mass dimorphism and tested each hypothesis (see Supplementary Table 1), we found the strongest support for the mating competition intensity hypothesis (H1). Sexual dimorphism in *f*_o_ increased during evolutionary transitions towards polygyny (*t* = −3.97; *p* < 0.001) and increased with a greater female-biased adult sex ratio (*t* = −2.09; *p* = 0.044). While some results are in the predicted direction, we found no clear statistical support (*p* > 0.050) for hypotheses H2-H4 in these models (Supplementary Table 1). In contrast to the prediction under the habitat hypothesis (H5), arboreal species exhibited lower *f*_o_ dimorphism compared to terrestrial ones (*t* = 2.17; *p* = 0.038; *p* = 0.093 with Tukey’s test for post hoc comparison). While a difference in *f*_o_ dimorphism was observed between arboreal species and species that are both arboreal and terrestrial (*t* = 4.84; *p* < 0.001), no clear difference was observed between terrestrial species and those that are both arboreal and terrestrial (*t* = 1.01; *p* = 0.578). Our coding of baboons as arboreal and terrestrial may have reduced our statistical power to detect differences in *f*_o_ dimorphism between habitats. In a post hoc test comparing *f*_o_ dimorphism between arboreal and terrestrial species (combining those that are terrestrial, and arboreal and terrestrial), arboreal species exhibited lower *f*_o_ dimorphism (*t* = 3.68; *p* < 0.001).

Multiple measures of mating competition intensity may correlate and influence sperm competition. Likewise, our measures of group size and social organization structures are likely interdependent. In various candidate models (Supplementary Table 2), we simultaneously tested multiple measures of mating intensity, with and without testes size [mating competition intensity hypothesis (H1) + sperm competition hypothesis (H4)], and group size and social organization structures [group size hypothesis (H2) + social organization hypothesis (H3)] against *f*_o_ dimorphism. In these models, we found support for the mating competition intensity hypothesis (H1) and group size hypothesis (H2). Specifically, *f*_o_ dimorphism increased with female-biased sex ratios (*t* = −2.15; *p* = 0.039) and group size (*t* = −2.43; *p* = 0.021). Although predicted under H1 but not H3, *f*_o_ dimorphism was higher in single-male/multi-female groups than in multilevel groups (*t* = −2.06; *p* = 0.048).

### Stepwise models, model averaging, and phylogenetic path analyses

**Fig. 2:**
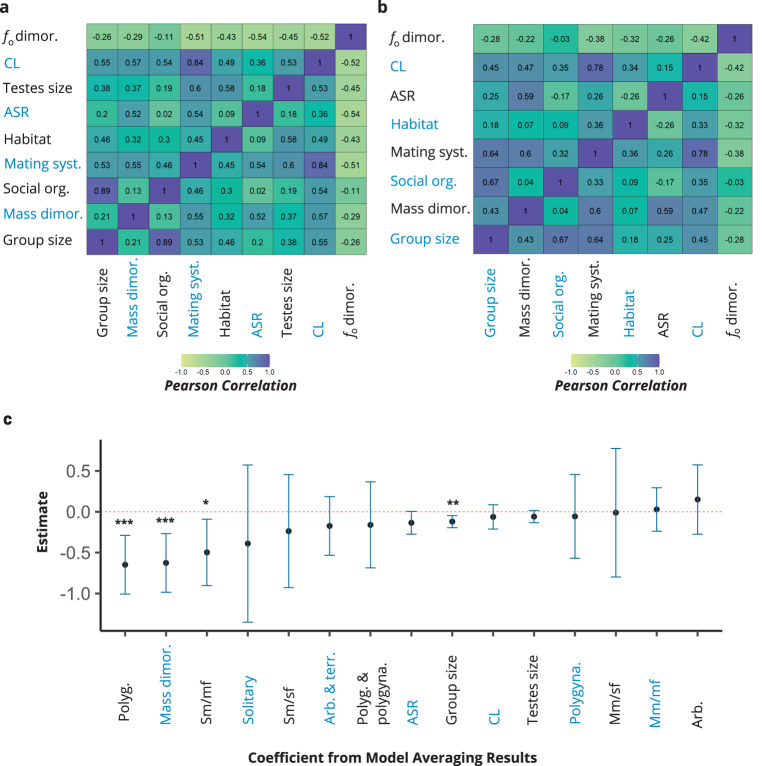
Correlations among study variables, and model-averaged parameter estimates across all tested models. Pearson correlation coefficients between pairs of variables are shown in heatmaps from models **a** without and **b** with control for the phylogenetic signal. Correlations that control for the phylogenetic signal were calculated using *corphylo* in *ape* R package. Testes size was dropped in the correlation analyses that control for the phylogenetic signal due to missing values. Panel **c** shows model averaging results with 95% confidence intervals calculated using *model.avg* in *MuMIn* R package. Monogamy was set as a reference level for the mating system, the terrestrial environment was set as a reference level for habitat, and multilevel society was set as a reference level for social organization. *P* values for model averaging results were calculated using two-tailed *z*-tests with no adjustment made for multiple comparisons; Polyg. (*p* < 0.001); dimor. (*p* < 0.001), Sm/mf (*p* = 0.017), Group size (*p* = 0.001). Note: dimor. dimorphism (male/female), CL competition level, ASR adult sex ratio (female/male), Syst. system, Org. organization, Sm/sf single-male/single-female, Mm/mf multi-male/multi-female, Sm/mf single-male/multi-female, Polygyna. polygynandrous, Polyg. polygynous, Arb. arboreal, Terr. terrestrial. For correlation analyses, three variables were recoded into continuous variables: habitat (arboreal = 1; arboreal and terrestrial = 2; terrestrial = 3), mating system (monogamy = −1; polygyny and polygynandry = 1), and social organization (solitary = 1; single-male/single-female = 2; multi-male/single-female = 3; single-male/multi-female = 3; multi-male/multi-female = 4; multilevel = 5). ^***^p < 0.001; ^**^p < 0.010. Source data are provided as a Source Data file.

However, these analyses depend on the selection of specific variables of interest, producing parameter estimates inferred from a limited set of candidate models. To reduce potential model selection bias and obtain more robust point and uncertainty estimates, we identified important predictors through stepwise-phylogenetic models (Supplementary Table 2) via *stepAIC* MASS R package^44^, and then averaged parameter estimates across all tested models. Using the natural average method^45^ via *model.avg* in *MuMIn* R package^46^, we reported model averaging results, with 95% confidence intervals. In our model averaging results (Fig. 2c), *f*_o_ dimorphism (i.e., lower male *f*_o_ values compared to females) increased during transitions towards polygyny (vs. monogamy) (*p* < 0.001), more female-biased sex ratios (*p* = 0.058), larger group size (*p* = 0.001), single-male/multi-female (vs. multilevel) group (*p* = 0.017), and increased body mass dimorphism (*p* < 0.001). Overall, these results (Supplementary Table 3) provide support for the mating competition intensity hypothesis (H1) and group size hypothesis (H2), but not for the social organization hypothesis (H3), sperm competition hypothesis (H4), or habitat hypothesis (H5). We incorporated uncertainty in phylogenetic relationships using 100 different trees from 10kTrees, which were produced from Bayesian phylogenetic methods. For each of the models tested above, we used model averaging procedures across 100 different models to estimate regression coefficients. These results produce statistically similar results (Supplementary Tables 4, 5) that are comparable to our main results with a consensus phylogeny. Lastly, we tested additional phylogenetic path analyses that incorporated the path that body size dimorphism evolved prior to the polygynous mating system. The phylogenetic path analysis that incorporated group size affecting *f*_o_ dimorphism yields weaker statistical support, which may be explained by reduced statistical power when analyses are restricted to species with monogamous and polygynous mating systems. Nevertheless, our analyses do not support the explanation that *f*_o_ dimorphism evolved before polygyny and instead favor the explanation that *f*_o_ dimorphism evolved in response to the polygynous mating system (Supplementary Fig. 2).

### Sexual selection and *f*_o_ dimorphism

Our results provide insight into the evolution of *f*_o_ dimorphism in primates (Fig. 1c). *f*_o_ dimorphism increased during evolutionary transitions towards larger group size, consistent with H2^5^. Rapid assessment of the status and threat potential of conspecifics may be critical in larger groups, where the reliability of social recognition is limited^5,35^, interaction among strangers is prevalent^47,48^, and physical confrontation is frequent^49,50^. Our data suggest that conspicuous secondary sexual characteristics such as low *f*_o_ evolved in males in such species.

Our results also suggest that a key driver of *f*_o_ dimorphism has been sexual selection via male contests. Female-biased sex ratios and polygynous mating system predicted increased *f*_o_ dimorphism. Adult sex ratio is a continuous (rather than categorical) measure of mating competition, with polygynous species having more female-biased sex ratios than monogamous and polygynandrous species, and has been recognized as an important factor in the evolution of mating systems, parental investment, and mating competition^51^. Similar to observations in birds^52^ and humans^53^, our results align with theory and data suggesting that males will tend to expend more mating effort when the sex ratio is female-biased and invest more parenting effort when the sex ratio is male-biased^51,54^. In species with intense male mating competition, such as gorillas and hamadryas baboons, the male mating effort is directed towards excluding same-sex rivals from social groups, leading to female-biased sex ratios in group composition. However, a recent study^55^ suggests that adult sex ratio is a cause, but not a consequence, of intense intrasexual selection among males; higher male mortality rates across amniotes (reptiles, birds, and mammals) may lead to female-biased sex ratios, which may, in turn, produce relatively larger body size in males to increase competitiveness for mating opportunities.

Similarly, female-biased sex ratio in our study is associated with greater body mass dimorphism (*r* = 0.52; *p* < 0.001) and competition level in males (*r* = 0.36; *p* = 0.027) (see Fig. 2 for other variables). Additionally, mating system predicted sex ratios (one-way ANOVA: *f* = 21.88, *p* < 0.001), with polygynous species having greater female-biased adult sex ratios than both monogamous (*p* < 0.001) and polygynandrous (*p* = 0.043) species (Benjamini–Hochberg corrections). In models that simultaneously included multiple measures of mating intensity, female-biased sex ratios more strongly predicted *f*_o_ dimorphism than did mating system, competition level, and body mass dimorphism. Therefore, the adult sex ratio may quantify mating competition intensity more precisely than do other measures. However, adult sex ratio did not remain a substantial predictor (*p* = 0.058) in our model-averaging results. This is likely due to biased estimates of adult sex ratio, which may weaken its relationship with *f*_o_ dimorphism. Our overall findings conform to the pattern that female-biased sex ratio favors increased male resource allocation to mating competition, leading males to delay sexual maturity in preparation for developing morphological and behavioral traits that facilitate mating competition^55,56^. These findings are also consistent with research on humans. The opportunity for sexual selection in human males increases with more female-biased sex ratios across small-scale societies^57^, and female-biased sex ratios predict increased male aggressiveness in the Standard Cross-Cultural Sample, even after controlling for geographical region, political complexity, and warfare mortality differences across societies^58^.

### Little support for the sperm competition hypothesis

Alternatively, male-biased adult sex ratios may reflect more intense sperm competition than female-biased adult sex ratios. Species that live in groups with male-biased sex ratios may have reduced *f*_o_ dimorphism because of a potential trade-off between investment in pre-copulatory and post-copulatory traits^38,39^ (e.g., investment in testes size is expected to reduce vocal tract length, and vice versa). However, we found no relationship between testes size and *f*_o_ dimorphism (H4). At least in humans, when the sex ratio is male-biased, females show more demanding preferences for male attractiveness^59^. Thus, if lower male *f*_o_ is selected primarily via mate choice, then increased *f*_o_ dimorphism should be present in species with more male-biased sex ratios. However, our findings suggest that *f*_o_ dimorphism decreased in species with more male-biased sex ratios and thus that male *f*_o_ may function more effectively in male contests than in female mate choice.

### Little support for the habitat hypothesis

Lastly, we found little support for the habitat hypothesis (H5) that arboreal species should exhibit more pronounced *f*_o_ dimorphism. Instead, we found that arboreal species have lower *f*_o_ dimorphism than terrestrial species. Although these findings contradict the prediction that overall *f*_o_ dimorphism evolves predominantly as a result of male vocalizations being selected for long-distance transmission, they are consistent with increased male–male competition in terrestrial species relative to arboreal ones^60,61^. Both predation pressure in terrestrial species and the body size restriction associated with the use of terminal branches during foraging in arboreal species have likely resulted in increased body size in terrestrial species and decreased body size in arboreal species^3,62^. Male body size is expected to be positively correlated with body size dimorphism (Rensch’s rule), and sexual selection explains this allometry^63^. Along with larger female group size in terrestrial species^3^, male–male competition would have become more important in terrestrial species relative to arboreal ones^52^, increasing sexual dimorphism in body size^62^, canine teeth^5^, and vocal fundamental frequency^1^, as observed in this study.

Comparative work in mammals^40^ also suggests that higher vocal frequencies in forest environments are useful for avoiding predation, locating prey, and maintaining social cohesion. Thus, higher-frequency vocal signals may be selected in both sexes in arboreal species.

### Body size and *f*_o_ dimorphism

Although body size and strength are key determinants of fighting ability across species including humans^64,65^, some sexually dimorphic traits such as low frequency or low-*f*_o_ vocalizations do not appear to be lower than expected for body size in species with greater sexual size dimorphism^11^ and may signal threat potential, aggressive intent, and underlying condition^25,66,67^. Examining sexual size dimorphism in relation to *f*_o_ dimorphism between males and females, not just in relation to *f*_o_ in males^11^_,_ is important as it better captures the extent to which selection pressures differ by sex and hence is likely to have been sexually selected. Additionally, sexual size dimorphism, an index that is often used to indicate male mating competition, is strongly influenced by phylogeny and ecological factors^68,69^. When mating opportunities are secured mostly through direct fighting, males would receive less benefit from vocal size exaggeration and obtain increased fitness by investing in larger body size that is useful in contests. Conversely, when the cost of body size or fights is high or female choice significantly affects male fitness^1^, visual displays and acoustic threats may be most effective. Our results suggest that deep male vocalizations in primates may have been driven by selection to win mating opportunities while avoiding costly fights by intimidating other males and/or attracting females.

In conclusion, our results highlight the likely influence of sexual selection in the origins and maintenance of sexual dimorphism in vocalization *f*_o_ across anthropoids, shedding light on the strength and mechanisms of sexual selection in humans and other primates. We suggest that sexual selection for deep male vocalizations may have been enhanced by larger group sizes, which favor rapid recognition of threat potential and status in male contest competition and possibly female mate choice. Future research should expand these comparative analyses to include vocal tract resonance frequencies, which have been linked to mating competition, size exaggeration, and mate choice in many species^11,14^.

## Methods

No ethical approval was obtained for this study, as the study used only existing data and datasets that are publicly available online. Data generated in this study are provided in the Supplementary Information and Source Data file.

We collected recordings of nonhuman primate calls through our own fieldwork, by contacting other primatologists, searching online databases such as the Macaulay Library (http://macaulaylibrary.org/), and combining these recordings with others from a previous study^1^. From these, we chose 2129 that were free from substantial background noise and produced by a single adult individual of known species and sex (*n* = 56 species; Supplementary Data 1). Species without recordings of at least two vocalizations from each sex were excluded [female: mean = 30.73, SD = 39.62, and range = (2–181); male: mean = 21, SD = 29.31, and range = (2–156) for the number of vocalizations], resulting in a database of 1914 recordings from 37 species. Rather than select calls thought to be analogous across species or produced by both sexes, we followed previous work^1^ in measuring vocalizations across all available call types (Supplementary Data 2) to maximize our ability to capture information about the physical properties of the sound source, such as vocal fold length and thickness. Comparisons of a single call type or group of types would complicate cross-species comparisons, as it is unclear whether call types are truly comparable across species. In some primate species, such as orangutans, the repertoire shared between males and females can be limited, occasionally down to one call type. Our sampling procedures yielded a large sample size from diverse sources, which should reduce bias due to random sampling.

Using the acoustic analysis software PRAAT v. 6.1.53, we measured *f*_o_ from each file (.wav or .aiff) by identifying in the raw waveform a segment in which cycles were clearly discernible (Fig. 1b). We then counted cycles along this segment (up to 20 cycles) and divided by the duration of the interval to calculate *f*_o_. We repeated this procedure for a second segment, if possible, and computed the mean *f*_o_ for each recording. Then, we averaged all other mean *f*_o_ values per sex to obtain separate male and female *f*_o_ averages for each species. Among files with two measurable segments of *f*_o_, the internal consistency between *f*_o_ measures for the two segments is high (*n* = 1431; Cronbach’s alpha = 0.97). We collected data on additional variables, such as body size, habitat, and mating system from online databases such as Animal Diversity Web http://animaldiversity.org/ and the published literature (Supplementary Data 1). To control for body size differences across species, we calculated body mass dimorphism for each species by dividing log_10_-transformed male body mass (in grams) by log_10_-transformed female body mass (in grams). We used mating system (monogamous, polygynandrous, or polygynous), adult sex ratios (number of adult females divided by number of adult males in breeding groups), and previously-published assessments^3^ of intrasexual competition level as measures of pre-copulatory sexual selection pressures. We used log_10_-transformed testes size as an index of sperm competition for post-copulatory sexual selection pressures. We also used log_10_-transformed mean group size of each species and categorized social organization as multi-male/multi-female, single-male/multi-female, single-male/single-female, multi-male/single-female, and multilevel groups. Then, we categorized the habitat as arboreal, terrestrial, or arboreal/terrestrial via information obtained from http://alltheworldsprimates.org. In addition to non-phylogenetic ordinary least-squares (OLS) models (two-sided tests), we conducted phylogenetically informed analyses using a consensus phylogeny^70^ (Fig. 1c). Except for a phylogenetic model with separate male and female *f*_o_ as dependent variables, which was analyzed using the R package MCMCglmm^71^, we assessed correlated evolution among our variables with phylogenetic generalized least-squares (PGLS) regression using the ape package, v. 5.0, in R^72^. We first conducted three PGLS models with the following correlation structures: Brownian motion, Grafen’s *ρ*, and Pagel’s *λ* to test the relationship between body size and mean *f*_o_ in females. The PGLS model with Grafen’s *ρ* produced the lowest Akaike Information Criterion statistic, and we used PGLS regression with Grafen’s *ρ* for all subsequent analyses to minimize Type 1 errors and to compare results consistently across models with a similar correlation structure. Grafen’s *ρ* close to zero indicates a weak phylogenetic signal, *ρ* < 1 indicates relatively more gradual recent evolution, and *ρ* > 1 indicates relatively faster recent evolution. Phylogenetic path analyses were conducted using the R package phylopath^73^.

### Reporting summary

Further information on research design is available in the Nature Portfolio Reporting Summary linked to this article.

## Supplementary information

Supplementary Information

Description of Additional Supplementary Files

Supplementary Data 1

Supplementary Data 2

Reporting Summary

## Data Availability

The vocal fundamental frequency data generated in this study have been deposited in the osf under 10.17605/OSF.IO/4WDUM and provided in the Supplementary Information and Source Data file. Primate data used in this study are available in the DRYAD database under 10.5061/dryad.r0160, and the primate phylogenetic data used in this study are available in the 10kTrees database under https://10ktrees.nunn-lab.org/downloadTrees.html. Source data are provided with this paper.

## Source data

Source Data

## Code availability

The analysis scripts for this study are available online at 10.17605/OSF.IO/4WDUM.
