# Supplementary material for: Group size and mating system predict sex differences in vocal fundamental frequency in anthropoid primates
Source: Nat Commun. 2023 Jul 10;14:4069. doi: 10.1038/s41467-023-39535-w (PMC10333282; doi:10.1038/s41467-023-39535-w)
Supplement: Supplementary file 1 — Supplementary Information [file 41467_2023_39535_MOESM1_ESM.pdf]

SUPPLEMENTARY INFORMATION

Group size and mating system predict sex differences in vocal fundamental frequency in anthropoid primates

Toe Aung[1,2], Alexander K. Hill[3], Dana Pfefferle[4], Edward McLester[5], James Fuller[6], Jenna M. Lawrence[6], Ivan Garcia-Nisa[7], Rachel L. Kendal[7], Megan Petersdorf[7], James P. Higham[8], Gérard Galat[9], Adriano R. Lameira[10], Coren L. Apicella[11], Claudia Barelli[12], Mary E. Glenn[13], Gabriel Ramos-Fernandez[14], and David A. Puts[1*]

[1]Department of Anthropology, Pennsylvania State University, University Park, PA, USA

[2]Psychology and Counseling Department, Immaculata University, Immaculata, PA, USA

[3]Department of Anthropology, University of Washington, Seattle, WA, USA

[4]Welfare and Cognition Group, Cognitive Neuroscience Laboratory, German Primate Center – Leibniz Institute for Primate Research, Goettingen, Germany & Leibniz-ScienceCampus Primate Cognition, German Primate Center & University of Goettingen, Goettingen, Germany

[5]Department for the Ecology of Animal Societies, Max Planck Institute of Animal Behavior, Konstanz, Germany

[6]Department of Ecology, Evolution, and Environmental Biology, Columbia University, NY, USA

[7]Department of Anthropology, Durham University, Durham, UK

[8]Department of Anthropology, New York University, 25 Waverly Place, New York, NY, USA

[9]IRD (French National Research Institute for Sustainable Development), Montpellier, France

[10]Department of Psychology, University of Warwick, Coventry, UK

[11]Department of Psychology, University of Pennsylvania, Philadelphia, PA, USA

[12]Department of Biology, University of Florence, Sesto Fiorentino, Florence, Italy

[13]Department of Anthropology, California State Polytechnic University Humboldt, Arcata, CA, USA

[14]Institute for Research on Applied Mathematics and Systems and C3-Centro de Ciencias de la Complejidad, Universidad Nacional Autonoma de Mexico, Mexico, Mexico City, Mexico

[*]Correspondence to D.A.P. (ORCID ID: 0000-0002-6478-1980)

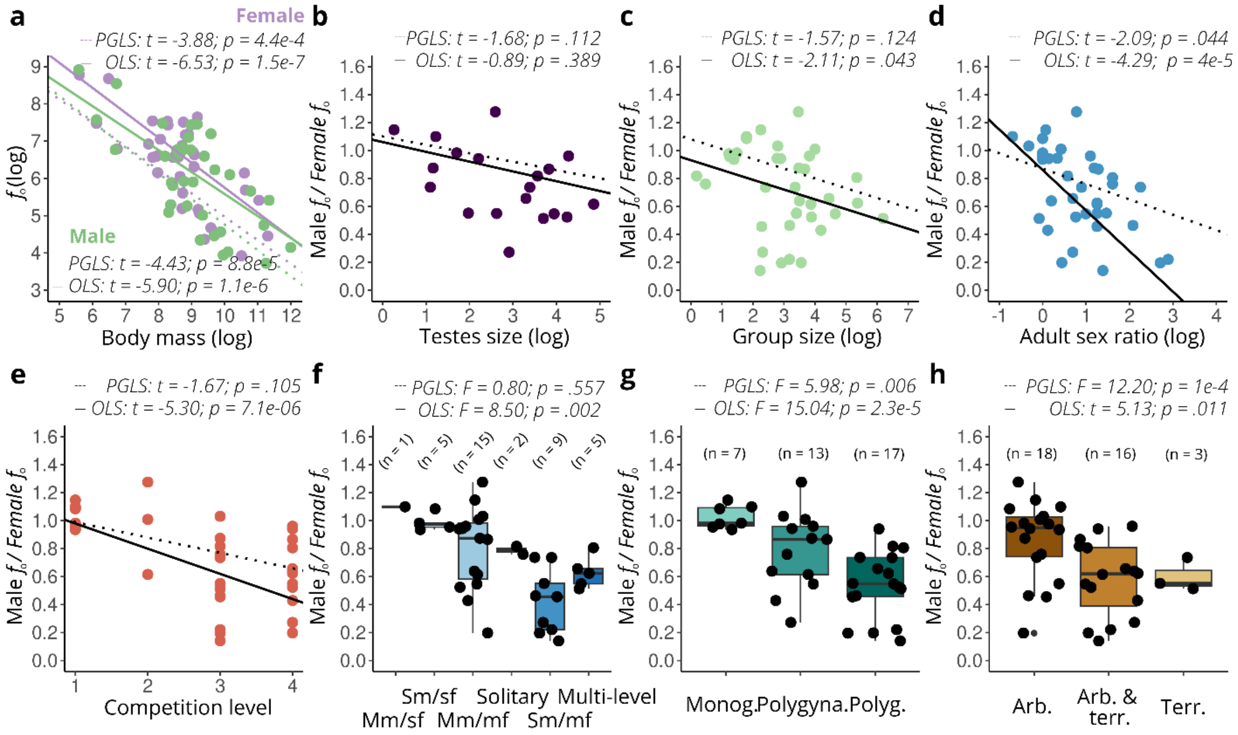

Supplementary Figure 1. Phylogenetic generalized least-squares (PGLS) and ordinary least-squares (OLS) regression models predicting evolutionary changes in f_o or f_o dimorphism. Panel (a) shows relationships between f_o and body mass for both sexes. f_o dimorphism is tested against (b) testes size, (c) group size, (d) adult sex ratio (female/male), (e) competition level, (f) social organization, (g) mating system, and (h) habitat in each model while controlling for body mass dimorphism and differences in the number of recording available for each species. *P* values were calculated using two-tailed *t*-tests (a-e), or one-tailed *F*-tests (f-h), with no adjustment made for multiple comparisons. The median value is represented by a bold central line, the interquartile range is depicted by boxed areas, and the minimum and maximum values are shown by the whiskers (f-h). Mm/sf = multi-male/single-female; Sm/sf = single-male/single-female; Mm/mf = multi-male/multi-female; Sm/mf = single-male/multi-female; Monog. = monogamous; Polygyna. = polygynandrous; Polyg. = polygynous; Arb. = arboreal, Terr. = terrestrial. Source data are provided as a Source Data file.

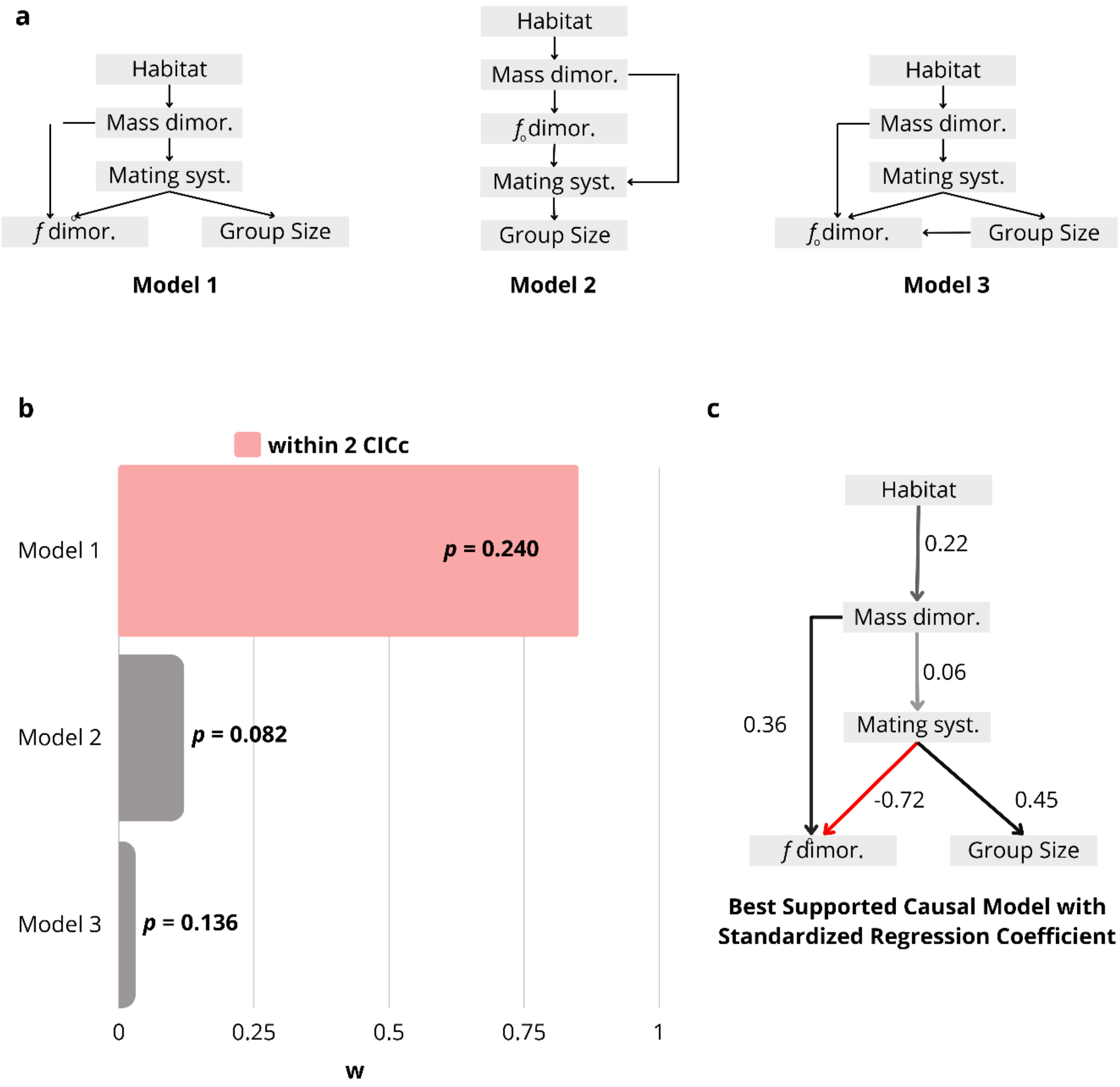

Supplementary Figure 2. Phylogenetically corrected path analysis models. Panel (a) shows the confirmatory models which describe the hypothesis that f_0 dimorphism evolved in response to polygynous mating system, prior to polygynous mating system, and in response to both polygynous mating system and group size. Panel (b) shows the relative importance of three causal models. *P* values were calculated using one-tailed *C*-tests, with no adjustment made for multiple comparisons. Panel (c) shows best supported causal model, with the standardized path coefficients. Source data are provided as a Source Data file.

Supplementary Table 1. Tests of individual hypotheses in phylogenetic generalized least-squares regression models predicting evolutionary changes in f_o dimorphism (gVIF df < 2).

Hypothesis	*Model*	*AIC*	*L.ratio*	R^2	ρ	*t*	*p*
**H1: mating competition intensity**	model[a] (*n* = 37)[+]	31.58	10.67	0.31	0.08		0.014
	polyg. vs. monog.					-1.98	0.057
	polygyna. vs. monog.					-0.91	0.368
	male/female mass (log)					-0.22	0.828
	model[b] (*n* = 24)	14.33	11.94	0.39	0.06		0.003
	polyg. vs. monog.					-3.97	7e-4
	male/female mass (log)					2.11	0.047
	model[c] (*n* = 37)	34.10	1.98	0.13	0.13		0.371
	polyg. & polygyna. vs. monog.					-0.69	0.495
	male/female mass (log)					-0.87	0.389
	model[d] (*n* = 37)	34.40	4.04	0.21	0.17		0.133
	competition level					-1.67	0.105
	male/female mass (log)					-0.21	0.834
	model[e] (*n* = 37)	35.47	2.83	0.15	0.16		0.243
	competition level					-1.23	0.229
	male/female mass (log)					-0.26	0.800
	model (*n* = 37)	33.57	5.79	0.19	0.09		0.055
	adult sex ratios					-2.09	0.044
	male/female mass (log)					-0.89	0.382
**H2: group size**	model (*n* = 37)	35.37	4.07	0.20	0.12		0.131
	group size (log)					-1.58	0.124
	male/female mass (log)					-1.46	0.154
**H3: social organization**	model (*n* = 37)[++]	43.70	4.55	0.21	0.10		0.602
	mm/mf vs. multi-level					0.74	0.463
	mm/sf vs. multi-level					0.50	0.623
	sm/mf vs. multi-level					-0.47	0.641
	sm/sf vs. multi-level					0.74	0.464
	solitary vs. multi-level					0.32	0.752
	male/female mass (log)					-0.36	0.723
**H4: sperm competition**	model (*n* = 19)	12.24	7.65	0.37	0.01		0.022
	testes size					-1.68	0.112
	male/female mass (log)					-2.21	0.042
**H5: habitat**	model (n = 37)[+++]	24.81	19.12	0.31	0.28		3e-04
	arb. vs. terr.					2.17	0.038
	arb. & terr. vs. terr.					-1.01	0.321
	male/female mass (log)					-0.34	0.736

[a]monog. tested against polyg. and polygyna. system; [b]monog. tested against polyg. only; [c]monog. tested against polyg. and polygyna. system combined; [d]humans treated as competition level 3; [e]humans treated as competition level 2. *P* values were calculated using two-tailed *t*-tests (a-e), with Tukey's HSD test used to account for multiple comparisons in post-hoc analyses. [+]Post-hoc comparisons (Tukey's test): polyg. vs. monog. (*p* = 0.106); polygyna. vs. monog. (*p* = 0.611); and polyg. vs. polygyna. (*p* = 0.007). [++]Post-hoc comparisons (Tukey's test) yield results that are not statistically different (p = 0.688 for sm/mf vs. mm/mf; *p* > 0.900 for all other comparisons). [+++]Post-hoc comparisons (Tukey's test: arb. vs. terr. (*p* = 0.074); arb. & terr. vs. terr. (*p* = 0.564); and arb. & terr. vs. arb. (*p* = 1e-4). mm/sf = multi-male/single-female; sm/sf = single-male/single-female; mm/mf = multi-male/multi-female; sm/mf = single-male/multi-female; monog. = monogamous; polygyna. = polygynandrous; polyg. = polygynous; arb. = arboreal, terr. = terrestrial.

Supplementary Table 2. Simultaneous tests of multiple hypotheses in phylogenetic generalized least-squares regression models predicting evolutionary changes in f_o dimorphism (gVIF df < 2).

Hypothesis	*Model*	*AIC*	*L.ratio*	R^2	ρ	*t*	*p*
**H1: mating competition intensity + H4: sperm competition**	model[a*] (n = 19)	23.79	16.38	0.41	0.01		0.012
	polyg. vs. monog.					-0.80	0.441
	polygyna. vs. monog.					0.24	0.814
	competition level					-0.28	0.786
	adult sex ratios					-0.71	0.491
	testes size (log)					-1.70	0.114
	male/female mass (log)					0.40	0.695
	model[b*] (n = 12)	15.91	15.86	0.73	0.01		0.007
	polyg. vs. monog.					-0.77	0.469
	competition level					-0.35	0.735
	adult sex ratios (log)					-1.24	0.261
	testes size (log)					-1.71	0.137
	male/female mass (log)					1.21	0.272
	model[c*] (n = 19)	24.35	9.24	0.36	0.10		0.114
	polyg. & polygyna. vs. monog.					-0.73	0.481
	competition level					0.07	0.945
	adult sex ratios (log)					0.88	0.392
	testes size (log)					-0.54	0.597
	male/female mass (log)					-1.22	0.243
**H1: mating competition intensity**	model[a^] (n = 37)	39.03	16.18	0.31	0.06		0.006
	polyg. vs. monog.					-0.75	0.461
	polygyna. vs. monog.					0.11	0.912
	competition level					-1.53	0.135
	adult sex ratios (log)					-1.73	0.093
	male/female mass (log)					0.79	0.432
	model[b^] (n = 24)	17.36	22.89	0.61	0.01		1e-4
	monog. vs. polyg.					-1.95	0.067
	competition level					-0.88	0.388
	adult sex ratios (log)					-3.25	0.004
	male/female mass (log)					3.15	0.005
	model[c^] (n = 37)	38.84	9.24	0.32	0.17		0.055
	polyg. & polygyna. vs. monog.					0.53	0.601
	competition level					-1.92	0.064
	adult sex ratios (log)					-2.17	0.038
	male/female mass (log)					0.30	0.769
**H2: group size + H4: social organization**	model (n = 37)	44.28	11.91	0.37	0.04		0.104
	group size (log)					-2.46	0.020
	mm/mf vs. multi-level					-0.45	0.654
	mm/sf vs. multi-level					-0.37	0.714
	sm/mf vs. multi-level					-2.06	0.048
	sm/sf vs. multi-level					-0.75	0.457
	solitary vs. multi-level					-0.82	0.416
	male/female mass (log)					-0.80	0.429
**Step-wise AIC**	model[d*] (n = 19)	-6.42	17.03	0.54	0.01		7e-4
	group size (log)					-3.45	0.003

male/female mass (log)					-3.70	0.002
model[e*] (n = 37)	11.17	29.67	0.29	0.01		2e-4
group size (log)					-2.04	0.052
polyg. vs. monog.					-0.75	0.459
polygyna. vs. monog.					-0.17	0.868
arb. vs. terr.					-1.38	0.180
arb. & terr. vs. terr.					-3.23	0.003
mm/mf vs. multi-level					0.22	0.917
mm/sf vs. multi-level					-0.03	0.917
sm/mf vs. multi-level					-2.52	0.126
sm/sf vs. multi-level					-0.71	0.100
solitary vs. multi-level					-0.83	0.087
adult sex ratios (log)					1.34	0.191

[a]monog. tested against polyg. and polygyna. system; [b]monog. tested against polyg. only; [c]monog. tested against polyg. and polygyna. system combined; [d]step-wise model without categorical mating systems; [e]step-wise model without testes values. *P* values were calculated using two-tailed *t*-tests with no adjustment made for multiple comparisons. mm/sf = multi-male/single-female; sm/sf = single-male/single-female; mm/mf = multi-male/multi-female; sm/mf = single-male/multi-female; monog. = monogamous; polygyna. = polygynandrous; polyg. = polygynous; arb. = arboreal, terr. = terrestrial.

Supplementary Table 3. Model averaging results for each variable predicting evolutionary changes in f_o dimorphism.

Model	*b*	*SE*	*Adj. SE*	*z*	*p*	CI.min	CI.max
polyg. vs. monog.	-0.65	0.17	0.18	3.53	4e-4	-1.01	-0.29
male/female mass (log)	-0.63	0.17	0.18	3.42	0.001	-0.98	-0.27
sm/mf vs. multi-level	-0.50	0.20	0.21	2.40	0.017	-0.90	-0.09
solitary vs. multi-level	-0.39	0.47	0.49	0.79	0.428	-1.35	0.57
sm/sf vs. multi-level	-0.24	0.34	0.35	0.67	0.501	-0.93	0.45
arb. & terr. vs. terr.	-0.17	0.18	0.18	0.95	0.343	-0.53	0.19
polyg. & polygyna. vs. monog.	-0.16	0.25	0.27	0.60	0.549	-0.69	0.37
adult sex ratios (log)	-0.14	0.07	0.07	1.89	0.058	-0.27	0.00
group size (log)	-0.12	0.03	0.04	3.20	0.001	-0.19	-0.05
competition level	-0.06	0.07	0.08	0.83	0.408	-0.21	0.09
testes size (log)	-0.06	0.04	0.04	1.55	0.120	-0.13	0.02
polygyna. vs. monog.	-0.06	0.25	0.26	0.22	0.827	-0.57	0.46
mm/sf	-0.01	0.38	0.40	0.03	0.977	-0.80	0.78
mm/mf	0.03	0.13	0.14	0.21	0.837	-0.24	0.29
arb. vs. terr.	0.15	0.21	0.22	0.69	0.488	-0.27	0.57

[a]monogamy tested against polygyny and polygynandrous system; [b]monogamy tested against polygyny only; [c]monogamy tested against polygyny and polygynandrous system combined; [d]humans treated as competition level 3; [e]humans treated as competition level 2. *P* values were calculated using two-tailed *z*-tests with no adjustment made for multiple comparisons. mm/sf = multi-male/single-female; sm/sf = single-male/single-female; mm/mf = multi-male/multi-female; sm/mf = single-male/multi-female; monog. = monogamous; polygyna. = polygynandrous; polyg. = polygynous; arb. = arboreal, terr. = terrestrial.

Supplementary Table 4. Model averaging results for tests of individual hypotheses in phylogenetic generalized least-squares regression models with 100 different phylogenetic trees predicting evolutionary changes in f_o dimorphism (VIF< 2).

Hypothesis	*Model*	*b*	*SE*	*Adj. SE*	*z*	*p*
**H1: male contest competition**	model[a] (*n* = 37)					
	polyg. vs. monog.	-0.43	0.21	0.22	1.98	0.048
	polygyna. vs. monog.	-0.19	0.19	0.20	0.95	0.341
	male/female mass (log)	0.06	0.23	0.24	0.25	0.803
	model[b] (*n* = 24)					
	polyg. vs. monog.	-0.66	0.17	0.18	3.74	1e-4
	male/female mass (log)	0.42	0.20	0.21	1.99	0.046
	model[c] (*n* = 37)					
	polyg. & polygyna. vs.	-0.16	0.22	0.23	0.72	0474
	male/female mass (log)	-0.19	0.23	0.24	0.80	0.426
	model[d] (*n* = 37)					
	competition level	-0.11	0.07	0.07	1.74	0.083
	male/female mass (log)	-0.03	0.23	0.24	0.14	0.892
	model[e] (*n* = 37)					
	competition level	-0.09	0.07	0.07	1.33	0.184
	male/female mass (log)	-0.04	0.25	0.26	0.16	0.871
	model (*n* = 37)					
	adult sex ratios	-0.11	0.05	0.05	2.07	0.038
	male/female mass (log)	-0.19	0.21	0.22	0.88	0.380
**H2: group size**	model (*n* = 37)					
	group size (log)	-0.07	0.04	0.05	1.54	0.123
	male/female mass (log)	-0.30	0.21	0.22	1.38	0.167
**H3: social organization**	model (*n* = 37)					
	mm/mf vs. multi-level	0.08	0.13	0.14	0.60	0.548
	mm/sf vs. multi-level	0.19	0.39	0.40	0.48	0.634
	sm/mf vs. multi-level	-0.08	0.15	0.16	0.53	0.593
	sm/sf vs. multi-level	0.24	0.32	0.34	0.70	0.482
	solitary vs. multi-level	0.18	0.60	0.63	0.29	0.769
	male/female mass (log)	-0.10	0.26	0.27	0.36	0.720
**H4: sperm competition**	model (*n* = 19)					
	testes size	-0.06	0.04	0.04	1.55	0.120
	male/female mass (log)	-0.45	0.21	0.22	2.04	0.041
**H5: habitat**	model (n = 37)					
	arb. vs. terr.	0.22	0.11	0.12	1.93	0.053
	arb. & terr. vs. terr.	-0.10	0.10	0.11	0.94	0.346
	male/female mass (log)	-0.08	0.19	0.20	0.40	0.686

[a]monog. tested against polyg. and polygyna. system; [b]monog. tested against polyg. only; [c]monog. tested against polyg. and polygyna. system combined; [d]humans treated as competition level 3; [e]humans treated as competition level 2. *P* values were calculated using two-tailed *z*-tests with no adjustment made for multiple comparisons. mm/sf = multi-male/single-female; sm/sf = single-male/single-female; mm/mf = multi-male/multi-female; sm/mf = single-male/multi-female; monog. = monogamous; polygyna. = polygynandrous; polyg. = polygynous; arb. = arboreal, terr. = terrestrial.

Supplementary Table 5. Model averaging results for simultaneous tests of multiple hypotheses in phylogenetic generalized least-squares regression models with 100 different phylogenetic trees predicting evolutionary changes in f_o dimorphism (VIF< 2).

Hypothesis	*Model*	*b*	*SE*	*Adj.*	*z*	*p*
**H1: mating competition intensity + H4: sperm competition**	model[a] (n = 19)					
	polyg. vs. monog.	-0.22	0.28	0.31	0.72	0.473
	polygyna. vs. monog.	0.08	0.31	0.35	0.22	0.829
	competition level	-0.02	0.07	0.07	0.25	0.803
	adult sex ratios	-0.08	0.11	0.12	0.64	0.523
	testes size (log)	-0.08	0.05	0.05	1.53	0.125
	male/female mass (log)	0.13	0.33	0.37	0.36	0.718
	model[b] (n = 12)					
	polyg. vs. monog.	-0.20	0.26	0.32	0.62	0.536
	competition level	-0.03	0.08	0.10	0.28	0.777
	adult sex ratios (log)	-0.13	0.10	0.13	0.99	0.320
	testes size (log)	-0.10	0.06	0.07	1.37	0.170
	male/female mass (log)	0.32	0.27	0.33	0.97	0.332
	model[c] (n = 19)					
	polyg. & polygyna. vs. monog.	-0.24	0.33	0.37	0.66	0.509
	competition level	-0.01	0.09	0.09	0.06	0.953
	adult sex ratios (log)	0.09	0.11	0.12	0.80	0.422
	testes size (log)	-0.03	0.05	0.06	0.51	0.612
	male/female mass (log)	-0.34	0.28	0.31	1.10	0.272
**H1: mating competition intensity**	model[a] (n = 37)					
	polyg. vs. monog.	-0.18	0.24	0.24	0.74	0.462
	polygyna. vs. monog.	0.03	0.22	0.22	0.12	0.908
	competition level	-0.11	0.06	0.07	1.59	0.112
	adult sex ratios (log)	-0.09	0.05	0.05	1.69	0.091
	male/female mass (log)	0.20	0.24	0.25	0.82	0.415
	model[b] (n = 24)					
	polyg. vs. monog.	-0.44	0.23	0.24	1.82	0.068
	competition level	-0.06	0.07	0.08	0.83	0.408
	adult sex ratios (log)	-0.15	0.05	0.05	3.05	0.002
	male/female mass (log)	0.56	0.18	0.19	2.95	0.003
	model[c] (n = 37)					
	polyg. & polygyna. vs. monog.	0.14	0.24	0.25	0.54	0.586
	competition level	-0.15	0.07	0.08	1.98	0.048
	adult sex ratios (log)	-0.11	0.05	0.05	2.10	0.036
	male/female mass (log)	0.08	0.23	0.24	0.35	0.729
**H2: group size + H4: social organization**	model (n = 37)					
	group size (log)	-0.16	0.06	0.06	2.48	0.013
	mm/mf vs. multi-level	-0.07	0.14	0.15	0.51	0.613
	mm/sf vs. multi-level	-0.16	0.40	0.42	0.37	0.710
	sm/mf vs. multi-level	-0.39	0.18	0.19	2.10	0.035
	sm/sf vs. multi-level	-0.29	0.36	0.37	0.78	0.433
	solitary vs. multi-level	-0.52	0.59	0.61	0.83	0.404
	male/female mass (log)	-0.20	0.23	0.24	0.83	0.408
**Step-wise AIC**	model[d] (n = 19)					
	group size (log)	-0.12	0.03	0.04	3.20	0.001

male/female mass (log)	-0.63	0.17	0.18	3.42	0.001
model[e] (n = 37)					
group size (log)	-0.13	0.06	0.06	1.95	0.052
polyg. vs. monog.	-0.19	0.26	0.28	0.72	0.474
polygyna. vs. monog.	-0.04	0.25	0.26	0.16	0.873
arb. vs. terr.	-0.19	0.14	0.15	1.31	0.190
arb. & terr. vs. terr.	-0.45	0.14	0.15	3.08	0.002
mm/mf vs. multi-level	0.03	0.13	0.14	0.21	0.837
mm/sf vs. multi-level	-0.01	0.38	0.40	0.03	0.977
sm/mf vs. multi-level	-0.50	0.20	0.20	2.40	0.017
sm/sf vs. multi-level	-0.24	0.34	0.35	0.67	0.501
solitary vs. multi-level	-0.39	0.47	0.49	0.79	0.428
adult sex ratios (log)	0.09	0.07	0.07	1.28	0.201

[a]monogamy tested against polygyny and polygynandrous system; [b]monogamy tested against polygyny only; [c]monogamy tested against polygyny and polygynandrous system combined; [d]step-wise model without categorical mating systems; [e]step-wise model without testes values. *P* values were calculated using two-tailed *z*-tests with no adjustment made for multiple comparisons. mm/sf = multi-male/single-female; sm/sf = single-male/single-female; mm/mf = multi-male/multi-female; sm/mf = single-male/multi-female; monog. = monogamous; polygyna. = polygynandrous; polyg. = polygynous; arb. = arboreal, terr. = terrestrial.
