# Supplementary material for: Group size and mating system predict sex differences in vocal fundamental frequency in anthropoid primates
Source: Nat Commun. 2023 Jul 10;14:4069. doi: 10.1038/s41467-023-39535-w (PMC10333282; doi:10.1038/s41467-023-39535-w)
Supplement: Supplementary file 2 — Description of Additional Supplementary Files [file 41467_2023_39535_MOESM2_ESM.pdf]

File name: Supplementary Data 1
Description: Supplementary data file used for phylogenetic analyses. Data include primate species (Species), social structures (Soc), competition level (CL), the average number of adult females (Adult.Female), the average number of adult males (Adult.Male), group size (Group_size), habitat environment (Habitat), mating system (Categ_Mat_Sys), total number of recordings (Total_Rec), number of individual females (Indiv_Females), number of female recordings (Fem_Rec), number of individual males (Indiv_Males), number of male recordings (Male_Rec), female fundamental frequency in Hertz (F_MeanF0_Hz), male fundamental frequency in Hertz (M_MeanF0_Hz), sexual dimorphism in fundamental frequency in Hertz (M_to_F_F0), female body mass in gram (F_Mass_g), male body mass in gram (M_Mass_g), male testes size in gram (Testes), and references (Refs).

File name: Supplementary Data 2
Description: Supplementary data file which shows the number of individuals and calls, and call descriptions by sex for each species.
