# Supplementary material for: Group size and mating system predict sex differences in vocal fundamental frequency in anthropoid primates
Source: Nat Commun. 2023 Jul 10;14:4069. doi: 10.1038/s41467-023-39535-w (PMC10333282; doi:10.1038/s41467-023-39535-w)
Supplement: Supplementary file 5 — Reporting Summary [file 41467_2023_39535_MOESM5_ESM.pdf]

nature portfolio

Corresponding author(s): David Puts

Last updated by author(s): Jun 7, 2023

# Reporting Summary

Nature Portfolio wishes to improve the reproducibility of the work that we publish. This form provides structure for consistency and transparency in reporting. For further information on Nature Portfolio policies, see our Editorial Policies and the Editorial Policy Checklist.

## Statistics

For all statistical analyses, confirm that the following items are present in the figure legend, table legend, main text, or Methods section.

| n/a | Confirmed | |
|---|---|---|
| ☐ | ☒ | The exact sample size (*n*) for each experimental group/condition, given as a discrete number and unit of measurement |
| ☐ | ☒ | A statement on whether measurements were taken from distinct samples or whether the same sample was measured repeatedly |
| ☐ | ☒ | The statistical test(s) used AND whether they are one- or two-sided<br>*Only common tests should be described solely by name; describe more complex techniques in the Methods section.* |
| ☐ | ☒ | A description of all covariates tested |
| ☐ | ☒ | A description of any assumptions or corrections, such as tests of normality and adjustment for multiple comparisons |
| ☐ | ☒ | A full description of the statistical parameters including central tendency (e.g. means) or other basic estimates (e.g. regression coefficient) AND variation (e.g. standard deviation) or associated estimates of uncertainty (e.g. confidence intervals) |
| ☐ | ☒ | For null hypothesis testing, the test statistic (e.g. *F*, *t*, *r*) with confidence intervals, effect sizes, degrees of freedom and *P* value noted<br>*Give P values as exact values whenever suitable.* |
| ☒ | ☐ | For Bayesian analysis, information on the choice of priors and Markov chain Monte Carlo settings |
| ☒ | ☐ | For hierarchical and complex designs, identification of the appropriate level for tests and full reporting of outcomes |
| ☐ | ☒ | Estimates of effect sizes (e.g. Cohen's *d*, Pearson's *r*), indicating how they were calculated |

*Our web collection on statistics for biologists contains articles on many of the points above.*

## Software and code

Policy information about availability of computer code

| | |
|---|---|
| Data collection | Acoustic analysis software PRAAT (v. 6.1.53) was used to extract fundamental frequency measures from audio recordings. |
| Data analysis | The following R packages: ape (v. 5.6-2) , dplyr (v.1.1.0), geiger (v. 2.0.10), MASS (v. 7.3-58.2), MCMCglmm (v. 2.34), MuMIn (v. 1.47.1), nlme (v. 3.1-162), phylopath (v 1.1.3), phytools (v. 1.2-0), piecewiseSEM (v. 2.3.0), superheat (v. 0.1.0) were used for analysis. The analysis scripts are available online at https://osf.io/4wdum/?view_only=bdffea70f5064e268e69aaff77ccab04 |

For manuscripts utilizing custom algorithms or software that are central to the research but not yet described in published literature, software must be made available to editors and reviewers. We strongly encourage code deposition in a community repository (e.g. GitHub). See the Nature Portfolio guidelines for submitting code & software for further information.

## Data

Policy information about availability of data

All manuscripts must include a data availability statement. This statement should provide the following information, where applicable:

- Accession codes, unique identifiers, or web links for publicly available datasets
- A description of any restrictions on data availability
- For clinical datasets or third party data, please ensure that the statement adheres to our policy

The vocal fundamental frequency data generated in this study have been deposited in the osf under https://osf.io/4wdum/?

view_only=12e5084d7c1e4653a6d1cece72056426 and provided in the Supplementary Information and Source Data file. Primate data used in this study are available in the DRYAD database under https://doi.org/10.5061/dryad.r0160, and the primate phylogenetic data used in this study are available in the 10kTrees database under https://10ktrees.nunn-lab.org/downloadTrees.html

## Human research participants

Policy information about studies involving human research participants and Sex and Gender in Research.

| | |
|---|---|
| Reporting on sex and gender | n/a |
| Population characteristics | n/a |
| Recruitment | n/a |
| Ethics oversight | n/a |

Note that full information on the approval of the study protocol must also be provided in the manuscript.

# Field-specific reporting

Please select the one below that is the best fit for your research. If you are not sure, read the appropriate sections before making your selection.

☐ Life sciences ☐ Behavioural & social sciences ☒ Ecological, evolutionary & environmental sciences

For a reference copy of the document with all sections, see nature.com/documents/nr-reporting-summary-flat.pdf

# Ecological, evolutionary & environmental sciences study design

All studies must disclose on these points even when the disclosure is negative.

| | |
|---|---|
| Study description | Low-frequency male vocalizations may be favored by sexual selection because they intimidate rivals and/or attract mates. Sexual dimorphism in fundamental frequency (fo) may be more pronounced in species with more intense male mating competition and in those with large group size, where social knowledge is limited and efficient judgment of potential mates and competitors is crucial. These non-mutually exclusive explanations regarding the evolution of fo dimorphism have not been tested simultaneously across primate species. Therefore, in a sample of vocalizations (n = 1914 recordings) across 37 anthropoid species, we investigated whether fo dimorphism evolved in association with increased intensity of mating competition (H1), large group size (H2), multilevel social organization (H3), a trade-off against the intensity of sperm competition (H4), and/or poor acoustic habitats (H5), controlling for phylogeny and body size dimorphism. |
| Research sample | Following methods and data used in (Puts et al., 2016; https://royalsocietypublishing.org/doi/full/10.1098/rspb.2015.2830), we collected recordings of non-human primate calls through our own fieldwork, by contacting other primatologists, searching online databases such as the Macaulay Library (http://macaulaylibrary.org/), and combining with recordings and acoustic measures from (Puts et al., 2016; DOI: 10.1098/rspb.2015.2830). From these, we chose 2129 that were free from substantial background noise and produced by a single adult individual of known species and sex (n = 56 species). The sample is meant to represent species-and-sex typical fundamental frequency values of anthropoid primates. |
| Sampling strategy | No sample-size calculation was performed. Using the acoustic analysis software PRAAT v. 6.1.53, we measured fo from each file (.wav or .aiff) by identifying in the raw waveform a segment in which cycles were clearly discernible. We then counted cycles along this segment (up to 20 cycles) and divided by the duration of the interval to calculate fo. We repeated this procedure for a second segment, if possible, and computed mean fo for each recording. Then, we averaged all other mean fo values per sex to obtain separate male and female fo averages for each species. |
| Data collection | We collected recordings of non-human primate calls through our own fieldwork, by contacting other primatologists, searching online databases such as the Macaulay Library (http://macaulaylibrary.org/), and combining with recordings and acoustic measures reported from (Puts et al., 2016; DOI: 10.1098/rspb.2015.2830). Alexander K. Hill, Dana Pfefferle, Edward McLester, James Fuller, Jenna M. Lawrence, Ivan Garcia-Nisa, Rachel L. Kendal, Megan Petersdorf, James P. Higham, Gérard Galat, Adriano R. Lameira, Coren L. Apicella, Claudia Barelli, Mary E. Glenn, and Gabriel Ramos-Fernandez collected vocal recordings from the fieldwork. Toe Aung and Alexander K. Hill further extracted fundamental frequency measures and collected ecological variables from the published literature. |
| Timing and spatial scale | Recordings of vocalizations were collected by researchers in the course of their careers at different times. Between 2011 and 2014, the second author first contacted researchers for donating and contributing recordings of primate vocalizations, extracted fo measures, and reported the data (Puts et al., 2016; DOI: 10.1098/rspb.2015.2830). Between 2021 and 2022, the first author contacted additional researchers and collected new recordings from new researchers and online databases (Puts et al., 2016; DOI: 10.1098/rspb.2015.2830). The first author extracted fo measures for new recordings and combined with fo and other measures reported in Puts et al. (2016). The vocalizations were recorded from non-human primates found across South America, Africa, and Asia. |
| Data exclusions | Species without recordings of at least two vocalizations from each sex were excluded [female: mean = 30.73, SD = 39.62, and range = |

| | |
|---|---|
| Data exclusions | (2-181); male: mean = 21, SD = 29.31, and range = (2-156) for number of vocalizations], resulting in a database of 1914 recordings from 37 species. |
| Reproducibility | Among files with two measurable segments of fo, the internal consistency between fo measures for the two segments is high (n = 1431; Cronbach's alpha = 0.97). First segments of a randomly chosen files (n = 184; Puts et al., 2016) were re-measured to determine intra-measurer reliability, which was very high (Cronbach's α ~ 1.000). |
| Randomization | The study only considers correlated variables, and randomization is not applicable in this non-experimental study. |
| Blinding | The study only considers correlated variables, and the investigators' blinding regarding treatment/control group allocations during data collection is not relevant to this study. |

Did the study involve field work? ☒ Yes ☐ No

## Field work, collection and transport

| | |
|---|---|
| Field conditions | Recordings of vocalizations were collected by researchers in the course of their careers at different times. For the purpose of this study, we requested and collected recordings from researchers, along with accompanying information on call type. We do not have relevant information regarding field conditions, locations, access and disturbance. |
| Location | n/a |
| Access & import/export | n/a |
| Disturbance | n/a |

# Reporting for specific materials, systems and methods

We require information from authors about some types of materials, experimental systems and methods used in many studies. Here, indicate whether each material, system or method listed is relevant to your study. If you are not sure if a list item applies to your research, read the appropriate section before selecting a response.

### Materials & experimental systems

| n/a | Involved in the study | |
|---|---|---|
| ☒ | ☐ | Antibodies |
| ☒ | ☐ | Eukaryotic cell lines |
| ☒ | ☐ | Palaeontology and archaeology |
| ☐ | ☒ | Animals and other organisms |
| ☒ | ☐ | Clinical data |
| ☒ | ☐ | Dual use research of concern |

### Methods

| n/a | Involved in the study | |
|---|---|---|
| ☒ | ☐ | ChIP-seq |
| ☒ | ☐ | Flow cytometry |
| ☒ | ☐ | MRI-based neuroimaging |

## Animals and other research organisms

Policy information about studies involving animals; ARRIVE guidelines recommended for reporting animal research, and Sex and Gender in Research

| | |
|---|---|
| Laboratory animals | The study did not involve laboratory animals. |
| Wild animals | Only vocal calls were recorded by field researchers. No animals were captured, caught, or transported. |
| Reporting on sex | Findings apply to both sexes, and sex was considered in the study design. The vocalizer sex is coded based on reports from field researchers and online database description. Data analyzed include male and female fundamental frequency values from 37 species. |
| Field-collected samples | The study did not laboratory work with samples collected from the field. |
| Ethics oversight | No ethical approval was obtained for this study, as the study only used existing data and datasets that are publicly available online. |

Note that full information on the approval of the study protocol must also be provided in the manuscript.
